# NADPH oxidase p47phox siRNA attenuates adventitial fibroblasts proliferation and migration in apoE(-/-) mouse

**DOI:** 10.1186/s12967-015-0407-2

**Published:** 2015-01-28

**Authors:** Fang Xu, Ying Liu, Lei Shi, Wei Liu, Li Zhang, Hongjing Cai, Jie Qi, Yong Cui, Weichen Wang, Yejia Hu

**Affiliations:** Department of Pathophysiology, Binzhou Medical University, Yantai, China; Affiliated Hospital, Binzhou Medical University, 661 Huangheer Road, Binzhou, 256603 China

**Keywords:** NADPH oxidase, p47phox, Adventitia fibroblasts, Atherosclerosis, ApoE(-/-)

## Abstract

**Background:**

Reactive oxide species (ROS) derived from NADPH oxidases is involved in atherosclerosis. However, as a key component of NADPH oxidase, how p47phox regulates NADPH oxidases activity, ROS production and adventitial fibroblasts (AFs) function remains unclear.

**Methods:**

p47phox in aortic arteries of apoE(-/-) mice fed with hyperlipid diet was detected by immunohistochemistry. NADPH oxidase activity, superoxide anion (O_2_^−^) generation and p47phox expression were analyzed in primary AFs treated by diphenyleneiodonium (DPI). The proliferation and migration of AFs were also analyzed.

**Results:**

p47phox expression was low in the aortic adventitia but high in the site of intimal injury with continuous hyperlipidic diet. Compared to AFs from wild-type mice, AFs derived from apoE(-/-) mice exhibited elevated NADPH oxidase activity, O_2_^−^ production and higher mRNA and protein levels of p47phox, correlated with increased capability of proliferation and migration. DPI inhibited NADPH oxidase activity and AFs proliferation and migration in a dose-dependent manner. In addition, siRNA mediated knockdown of p47phox attenuated the proliferation and migration of AFs derived from apoE(-/-) mice.

**Conclusion:**

p47phox plays a critical role in the regulation of adventitial fibroblast proliferation and migration and may be a new therapeutic target for neointimal hyperplasia.

## Background

Recent studies have implicated that in addition to smooth muscle cells, the fibroblasts proliferate and migrate from the vascular wall to the neointima in response to injury, mimicking one of the hallmark characteristics of early atherogenesis [1,2]. As the main cell type in the adventitia, adventitial fibroblasts (AFs) have been implicated a regulatory role in the vasculature upon injury or stress. In response to cytokines, injury, and stretch, AFs have been shown to be activated and undergo phenotypic and functional changes such as proliferation, differentiation, migration, upregulation of certain proteins expression, and release of factors that directly affect medial smooth muscle cell growth rhythm and stimulate the recruitment of inflammatory and progenitor cells to the vessel wall [3,4]. Reactive oxide species (ROS) derived from NADPH oxidases in adventitial fibroblasts appear to play an important role in this process [5,6].

NADPH oxidase is a well-characterized ROS-generating system that catalyzes the one-electron reduction of O_2_ to O_2_^−^, a precursor of a variety of other ROS. Initial generation of ROS by NADPH oxidases triggers the release of ROS from other sources [7]. NADPH oxidase is known to be a multicomponent enzyme complex that includes the two membrane-spanning polypeptide subunits p22phox and anchoring component gp91phox, which are associated with the plasma membrane cytoskeleton, along with three cytoplasmic polypeptide subunits, p40phox, p47phox and p67phox [8,9].

Previous studies have established the presence of four major components of classical NADPH oxidase, gp91phox, p22phox, p47phox, and p67phox in the perivascular region of rabbit and rat vessels and showed that p67phox is involved in adventitial fibroblast oxidase activity [10,11]. However, another study implicated that p47phox may play a greater role in adventitial fibroblast proliferation and neointimal hyperplasia than p67phox [12]. Therefore, the function of each subunit of NADPH oxidases in vascular tissues, and the precise manner in which NADPH oxidases activity is modulated remains debatable.

The apoE-deficient mice develop extensive atherosclerotic lesions and have been regarded as excellent model for atherosclerosis research [13]. In this study, we aimed to investigate the role of NADPH oxidase subunit p47phox in the hyperlipid diet induced atherosclerosis in apoE(-/-) mouse model and examine how p47phox level and NADPH oxidase activity are correlated with the function of adventitial fibroblasts during the development of atherosclerosis. In addition, we explored the possibility of gene therapy to target p47phox using siRNA strategy.

## Materials and methods

### Animals and preparation of mouse aortic AFs

C57BL/6 wild-type and AopE(-/-) mice were purchased from Peking University Health Science Center. Wild-type C57BL/6 mice were used as controls. Animal studies were approved by the Ethics Committee of Binzhou Medical University. AFs were prepared from 8-week-old C57BL/6 wild-type and apoE(-/-) mice fed a hyperlipidic diet (15%, w/w saturated fat, 0.25% cholesterol) for 2 weeks as described previously [14]. Briefly, aortas were aseptically removed from 8 mice and cleaned of fat tissue and blood cells. These aortas were pooled and cut into small pieces and then placed on tissue dishes in Dulbecco’s modified Eagle’s medium (DMEM) (Gibco-BRL, Gaithersburg, USA) containing 15% (v/v) fetal calf serum (FCS) (Gibco-BRL, Gaithersburg, USA). Fibroblasts grew from these explants within 10 days and then passaged for use. Fibroblasts between 3-7 passages were used in the following experiments.

### Cell culture and siRNA transfection

RNA duplexes of 21 nucleotides specific for mouse p47phox were designed based on mRNA sequence in gene bank. From the open reading frame of p47phox mRNA sequences, duplexes of the type AA (N19) UU were designed in order to obtain a 21-nt sense and 21-nt antisense strand with symmetric 2-nt 3′ overhangs of identical sequence. Three p47phox stealth siRNAs were selected and submitted to a BLAST search to avoid the targeting of other homologous genes and chemically synthesized and modified by Invitrogen (Shanghai, China). The sequences of three p47phox stealth siRNAs were MSS206954 sense: 5′-UUAACCAGGAACAUGUACACAUAGU-3′ and anti-sense: 5′-ACUAUGUGUACAUGUUCCUGGUUAA-3′; MSS206955 sense: 5′-UCAACAGCAGCGUACGCUUUGAUGG-3′ and anti-sense: 5′- CCAUCAAAGCGUACGCUGCUGUUGA-3′; MSS206956 sense: 5′-GGUCUCCUGGUAGAGUUGAUAAUCA-3′ and anti-sense: 5′-UGAUUAUCAACUCUACCAGGAGACC-3′. The one with best inhibitory effect was chosen for the following experiment. Non-interference siRNA sequences at the same position were designed as control.

AFs derived from apoE(-/-) at 5 × 10^5^ cell/ml were seeded into a 6-well plate with DMEM containing 10% FBS without antibiotics until 50-70% of confluence. 100 μM of p47phox stealth siRNA was transfected into cells by FuGENE after cells were starved for 24 h in DMEM without serum. The total RNA and protein were extracted for analysis of p47phox mRNA and protein levels, respectively. The cell lysates were used for analysis of NADPH oxidase activity.

### Immunohistochemistry analysis

Monoclonal mouse anti-p47phox was used to detect p47phox expression. Sections were heated at 60°C for 30 min, deparaffinized and hydrated using standard techniques, and then boiled in 10 mM citric acid buffer for 10 min for antigen retrieval. The sections were then incubated in 0.3% H_2_O_2_ in 80% methanol for 30 min to remove the endogenous perioxidase. Immunostaining was performed using a mouse IgG kit (Maixin Biotech, Fuzhou, China). Briefly, the sections were blocked with 10% normal horse serum for 30 min and then incubated overnight at 4°C with primary antibody (1:80 dilution in PBS containing 2% normal horse serum). Negative controls were incubated with the same concentration of matching IgG isotype (IgG2a). After washing with PBS, sections were incubated with biotinylated secondary antibody (1:400 dilution in PBS containing 2% normal horse serum) at 37°C for 15 min, followed by the incubation with HRP conjugated-streptavidin at 37°C for 15 min, and then developed with AEC (3-amino-9-ethylcarbazole) reagent at 37°C for 15 min and mounted in aqueous based mounting medium.

### Determination of NADPH oxidase activity

Cells in a 96-well microplate were made quiescent by serum starvation for 24 h and cultured for 24 h in DMEM containing 10% FBS in the absence or presence of 10 μM DPI. Cells were lysed by lysis buffer (GENMED, Shanghai, China). NADPH oxidase activity in cell lysate was determined by using the colorimetric method following the protocol. The absorbance at 550 nm was measured at 0 min and every 60 sec for 15 min in a UV/Vis photometer (BioPhotometer, Eppendorf, Westbury, NY, USA). The actual NADPH activity was calculated by subtracting the specific activity.

### Measurement of superoxide anion

The superoxide anion was determined by water soluble tetrazolium (WST-1) kit (Beyotime, Shanghai, China) following the instruction. Cells were seeded into 96-well microtiter plates at 5 × 10^3^ cells/well and cultured for 48 h. Then cells were made quiescent by serum starvation for 24 h and cultured for 24 h in DMEM containing 10% FBS. 10 μM of DPI was added to each well and incubated for an additional 24 h. The absorbance (A) at 450 nm was measured using a microplate reader. Each experiment was performed in triplicate.

### Cell proliferation assay

The proliferation of cells was examined with a CCK-8 kit (Dojindo Laboratories, Shanghai, China) following the instruction. Cells were seeded into 96-well microtiter plates with 5 × 10^3^ cells/well and cultured for 48 h. Then cells were made quiescent by serum starvation for 24 h and cultured for 24 h in DMEM containing 10% FBS in the absence or presence of various concentrations of DPI (0, 1, 5, 10, 20 μM). Ten μl of CCK-8 solution was added to each well and incubated for 1 h. The absorbance (A) at 450 nm was measured using a microplate reader. Each experiment was performed in triplicate.

### Cell migration assay

Cell migration was measured in a transwell chamber apparatus with 8 μm pore size and a polycarbonate membrane (Costar, New York, USA). Briefly, the cell suspension (1 × 10^4^ cells/ml) was loaded in the upper compartment of the chamber in the absence or presence of 10 μM DPI, whereas DMEM containing 20% FCS was added to the lower compartment of the chamber. The filters were maintained at 37°C in a humidified atmosphere containing 5% CO_2_. After 20 h, the filters were removed, and cells remaining on the upper surface of the membrane were removed with a cotton swab. Then the membranes were washed with PBS twice, and cells adhering beneath the membrane were fixed in 4% paraformaldehyde and counterstained with hematoxylin (Sigma). The migration of cells was quantified by cell counts of 5 random fields at 100x magnification in each membrane. Each experiment was performed in triplicate.

### RT-PCR analysis

Total RNA was extracted from the cultured cells with Trizol reagent (Invitrogen Inc., USA) in accordance with the manufacturer’s protocol. Reverse transcription reaction was performed and the products of cDNA were used as template for PCR. Primers used were as follows: p47phox forward: 5′-ACATCACAGGCCCCATCAT-3′ and reverse: 5′-ATGGATTGTCCTTTGTGCC-3′; β-actin forward: 5′-GGCATCGTGATGGACTCCG-3′ and reverse: GCTGGAAGGTGGACAGCGA. Amplification cycles were done at 95°C for 3 min, 52°C for 1 min, and 72°C for 1 min, with a final extension of 5 min at 72°C. PCR product lengths were 612 bp. PCR products were detected on 2% agarose gel by electrophoresis and ethidium bromide staining. Gels were scanned on a digital imaging system and the band intensity values were transformed logarithmically, and mRNA level was calculated as a ratio to that of β-actin. Results presented were representative of three independent experiments.

### Western blot analysis

Cells from cultures were washed three times in PBS and incubated in lysis buffer (50 mM Tris–Hcl pH 8.0, 150 mM NaCl, 0.1% SDS, 1% Nonidet P-40, 0.02% sodium azide, 100 μg/ml PMSF, 1 μg/ml peptin, and 1 μg/ml aprotinin) for 30 min on ice. The homogenate was centrifuged at 16,000 g for 45 min at 4°C. An equal amount of protein from each lysate was subjected to 12% SDS–PAGE and transferred onto a nitrocellulose membrane. After blocking for 1.5 h in 5% nonfat dried milk containing 0.1% Tween-20, the membrane was incubated at 4°C overnight with the following antibodies: rabbit polyclonal anti-p47phox (1:100, Santa Cruz, CA, USA). The membrane was washed and further incubated for 1 h at room temperature with HRP-conjugated secondary Abs. Following washing, immunoreactive bands were detected using chemiluminescence reagent (Amersham Biosciences).

### Statistical analysis

Results were expressed as mean ± SD. Significance was determined by Student’s *t*-test. Values of *P* < 0.05 was considered statistically significant.

## Results

### Immunohistochemical staining of p47phox in aortic arteries

**Figure 1:**
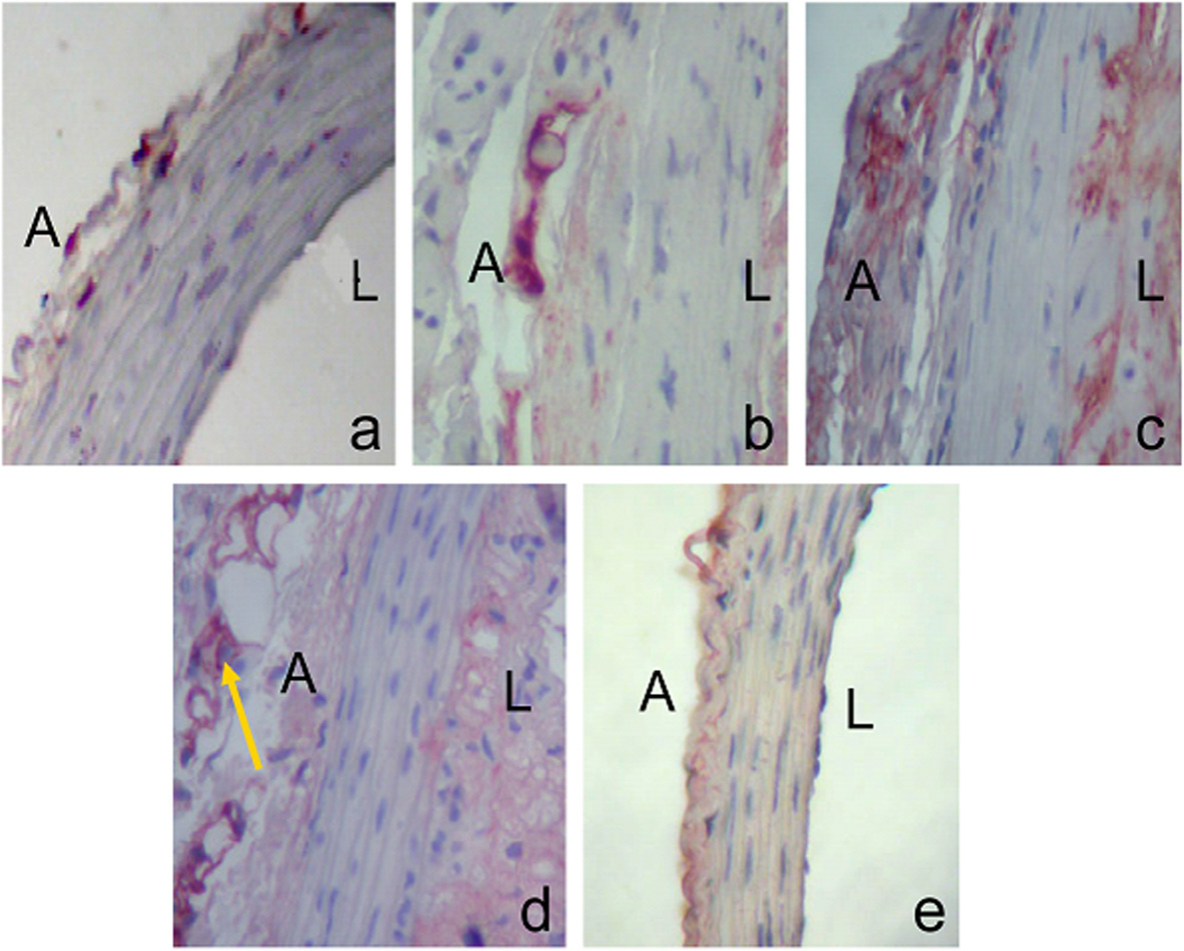
**Immunohistochemical staining of p47phox in the aorta section.** **a**. p47phox in aorta section from apoE(-/-) mouse with normal diet. **b**-**d**, p47phox in aorta section from apoE(-/-) mouse after hyperlipidic diet at 2 weeks **(** **b** **)**, 4 weeks **(** **c** **)** and 6 weeks **(** **d** **)**. **e**, p47phox in aorta section from C57BL/6 mouse with normal diet. A: adventitia; L: lumen. Magnification: X40.

There was low expression p47phox in the aortic arteries adventitia at 6-week old apoE(-/-) mice as shown in Figure 1a. The p47phox expression was increased and more cells became positive with continuous hyperlipidic diet and it started to present in the site of intimal injury after hyperlipid diet for 2 weeks and spread over time (Figure 1b-1d, Table 1). At 8 weeks after hyperlipidic diet, p47phox translocate to the cell membrane. A small amount of p47phox expression was observed in the aortic adventitia of C57BL/6 mice as well (Figure 1e). However, there were no changes at various time points (data not shown).

**Table 1:** **Expression of p47phox after hyperlipid diet in apoE(-/-) mice**

**Duration of hyper-lipid diet (weeks)**	**Adventitia**	**Intima**
0	(+)	(-)
2	(+)	(+)
4	(++)	(++)
8	(+++)	(+++)

Grades: (-): no stain; (+): <25% of positive cells; (++): 25%-50% positive cells.

### NADPH Oxidase activity and superoxide anion production in AFs

Oxidative stress is linked to the development of atherosclerosis in a variety of circumstances [15,16]. Here we sought to determine NADPH oxidase activity in AFs derived from hyperlipid induced apoE(-/-) mice. The AFs derived from apoE(-/-) mice exhibited higher NADPH oxidase activity than those from C57BL/6 wild type mice. The elevated production of superoxide anion was observed as well in apoE(-/-) mice compared to that in the wild type mice. The difference between two groups were statistically significant (*P* < 0.05, Table 2). These data indicated that AFs were subject to oxidative stress in apoE(-/-) mice fed with hyperlipid diet.

**Table 2:** **NADPH oxidase activity and O2** ^**-**^ **production in AFs**

**Source of AFs**	**DPI**	**NADPH oxidase activity**	**O**_**2**_^**−**^ **production**
	(μM)	(u/min/mg)	(OD450)
ApoE(-/-)	0	0.302 ± 0.114^Δ^*	0.235 ± 0.031^Δ^*
	10	0.072 ± 0.013	0.039 ± 0.007
C57BL/6	0	0.127 ± 0.019*	0.134 ± 0.012*
	10	0.041 ± 0.011	0.037 ± 0.006

Δ: P < 0.01 *vs.* C57BL/6 mice derived AFs; *: P < 0.05 *vs.* 10 μM DPI.

### Expression of p47phox was increased in AFs

**Figure 2:**
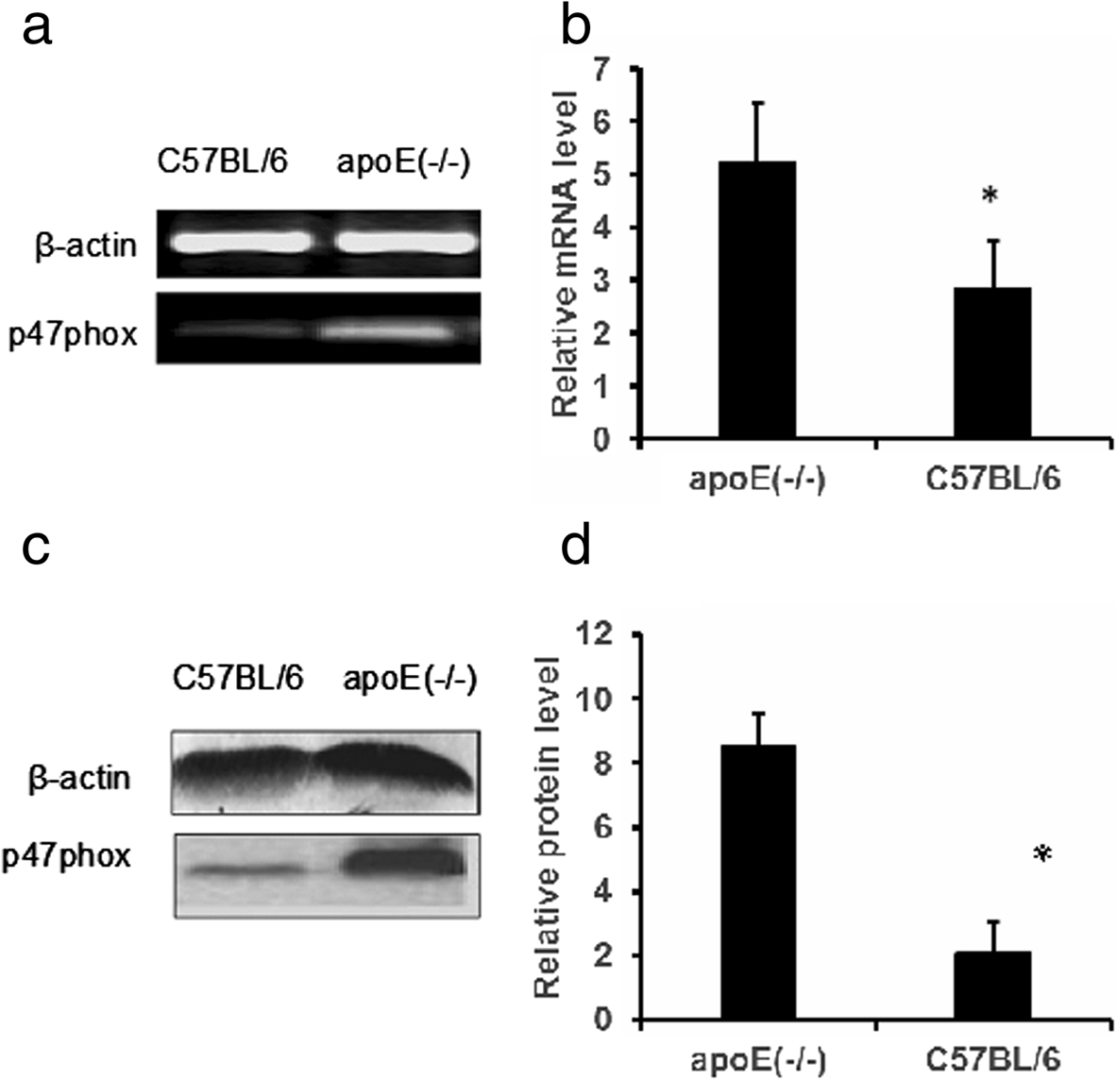
**The expression of p47phox in adventitial fibroblasts.** **a**. representative p47phox mRNA level in AFs analyzed by RT-PCR . **b** *,* the relative mRNA level of p47phox *vs.* β-actin in C56BL6 wild-type and apoE(-/-) mice. **c**, representative p47phox protein level in AFs analyzed by Western blot analysis. **d**, the relative protein level of p47phox *vs.* β-actin in C56BL6 wild-type and apoE(-/-) mice quantified by densitometry. *: P < 0.01 *vs.* C57BL/6 mice.

To understand the role of p47phox in the formation of atherosclerosis lesion in apoE(-/-) mice, we detected the mRNA and protein levels of p47phox in cultured primary AFs derived from apoE(-/-) mice. The results showed that both p47phox mRNA level (Figure 2a,b) and p47phox protein level (Figure 2c,d) were higher in AFs derived from apoE(-/-) mice than in AFs derived from wild-type mice, indicating that overexpressed p47phox in AFs might contribute to the enhanced NADPH activity.

### Effect of NADPH oxidase on AFs proliferation and migration

Our previous study showed that AFs derived from apoE(-/-) mice exhibited higher proliferation and migration compared to the wild-type mice [14]. Thus we speculated that the oxidative stress, in particular higher NADPH oxidase activity in AFs may inversely affect its proliferation and migration. To determine the role of NADPH oxidase activity in the proliferation and migration of AFs, we used diphenyleneiodonium (DPI), a specific inhibitor of NADPH oxidase, to monitor the characteristic changes of AFs proliferation and migration. The results showed that the production of superoxide anion was markedly reduced by addition of 10 μM DPI in both wild-type and apoE(-/-) mice, indicating that DPI inhibiting effect is NADPH oxidase specific. However, the magnitude of inhibition in AFs of aopE(-/-) was much more than that of wild-type mice (P < 0.05, Table 2).

**Figure 3:**
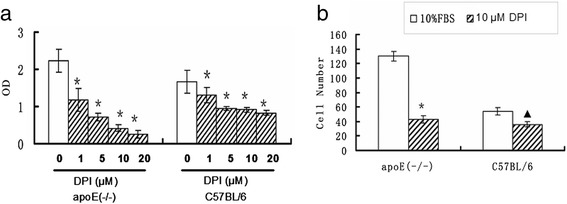
**DPI affects the proliferation and migration of AFs.** **a**. the proliferation of AFs in the absence or presence of various concentrations of DPI. *: *P* < 0.01 *vs* control AFs in the absence of DPI. **b**, the migration ability of AFs in the absence or presence of 10 μM DPI. *: *P* < 0.01 *vs* control AFs in the absence of DPI. ▲: P < 0.05 *vs* control AFs in the absence of 10 μM DPI.

Consistently with our previous study, AFs of aopE(-/-) exhibited higher capability of proliferation and migration compared to AFs from wild-type mice. As a result of the reduction of NADPH oxidase activity, the proliferation of AFs derived from both C57BL/6 and apoE(-/-) mice was inhibited in a DPI dose-dependent manner (Figure 3a). However, inhibitory effect on AFs proliferation derived from apoE(-/-) mice by 10 μM DPI was significantly more than that from wild-type C57BL/6. The migrations of AFs were also significantly inhibited in both mice groups but with higher magnitude in apoE(-/-) mice (*P* < 0.01) than that in C57BL/6 mice (P < 0.05) (Figure 3b). These results indicated that the higher NADPH oxidase activity positively correlated with the increased AFs capability to proliferate and migrate in apoE(-/-) mice.

### SiRNA-mediated knockdown of p47phox attenuates the NADPH oxidase activity, proliferation and migration of AFs in apoE(-/-)

**Figure 4:**
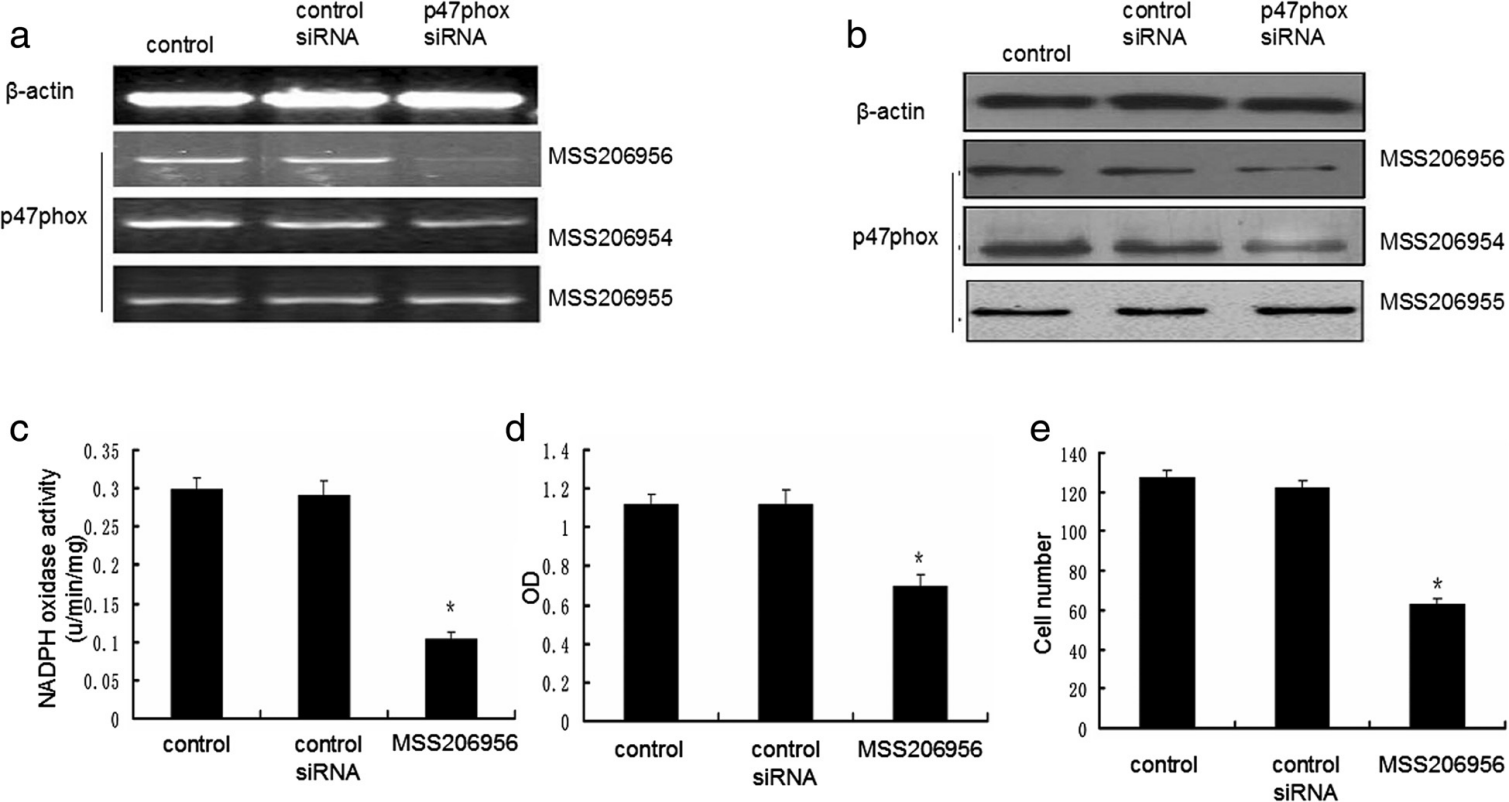
**p47phox siRNA inhibits NADPH oxidase activity, and the proliferation and migration of AFs from apoE(-/-) mice.** **a** *,* p47phox mRNA level in AFs transfected with different p47phox siRNAs. **b** *,* p47phox protein level in AFs transfected with different p47phox siRNAs. **c**, NADPH oxidase activity in AFs untreated, or treated with control siRNA or p47phox siRNA MSS206956. **d**, The proliferation of AFs untreated, or treated with control siRNA or p47phox siRNA MSS206956. **e**, The number of migrated AFs untreated, or treated with control siRNA or p47phox siRNA MSS206956. *: P < 0.01 *vs* control and control siRNA. Control: AFs without treatment; control siRNA: AFs transfected with non-interfering siRNA; MSS206956: AFs transfected with p47phox siRNA MSS206956.

To further determine the role of p47phox subunit in regulating the NADPH oxidase activity and characteristics of AFs, we utilized the siRNA strategy to knockdown p47phox. We designed three p47phox stealth siRNAs MSS206954, MSS206955, and MSS206956. The results showed that p47phox mRNA and protein levels were reduced up to 80% by MSS206956 compared to control siRNA, while the other two p47phox stealth siRNAs MSS206954 and MSS206955 had little effect to inhibit p47phox mRNA and protein levels (Figure 4a,b). Therefore, p47phox stealth siRNA duplexe MSS206956 was choosn for the following experiments. At 48 h post-transfection of p47phox siRNA MSS206956, the NADPH oxidase activity was reduced (Figure 4c) and the proliferation and migration ability of AFs were dramatically attenuated in cultured AFs (Figure 4d,e).

## Discussion

NADPH oxidase plays a critical role in vascular proliferative disorders [17,18]. Recent studies have gained substantial insights into the contribution of individual NADPH oxidase homologues to the maintenance of normal vascular function [19]. In smooth muscle cells and adventitial fibroblasts, critical components of NADPH oxidase are upregulated in response to vascular injury and atherosclerosis. In an apoE(-/-) murine model, it has been demonstrated that the total atherosclerosis lesions in the whole aorta in apoE(-/-) and p47phox (a key component of NADPH) double knockout mice with either normal or hyperlipid diet was decreased significantly compared to those in the apoE(-/-) mice, indicating that p47phox may be involved in development of atherosclerosis lesion formation [20].

The present study attempted to further clarify the role of NADPH oxidases subunit p47phox in the setting of atherosclerosis using apoE(-/-) murine model. Interestingly, we were able to show that p47phox level was increased in aortic adventitia in these mice fed with hyperlipid diet. P47phox started to present at the site of intimal injury and was found to translocate to the cell membrane with continuous hyperlipidic diet. However, the similar results were not observed in the wild type mice regardless of duration of hyperlipid diet. These data implicated that p47phox was activated and its expression pattern change might be an indicator of progression of hyperlipid induced atherosclerosis. And the certain type of cells expressing p47phox in adventitia may play an essential role in the development of atherogenesis.

These results were not consistent with some other studies showing that the depletion of leukocyte associated NADPH oxidase subunit had no effect on atherosclerosis lesion formation in apoE(-/-) mice with normal diet [21-23]. The discrepancies in the findings regarding the vascular consequences of p47phox^−/−^ could be related to the removal of p47phox, which causes immunodeficiency. These mice are prone to sub-clinical infections that are characterized by splenomegaly and inflammation which could counteract the anti-atherosclerotic effect of the loss of NADPH oxidase [19].

As the main cell type in adventitia, our previous study showed that adventitial fibroblasts were activated and exhibited functional changes such as proliferation and migration in the early stage of atherosclerosis [14]. ROS derived from NADPH oxidase appear to be involved in cell proliferation and migration [24,25]. Indeed, in a cultured system, primary AFs isolated from apoE(-/-) mice exhibited an elevated mRNA and protein levels of p47phox. Accordingly, NADPH oxidase activity and O_2_^−^ production in cultured AFs is significantly higher compared to the control. The pre-incubation of cells with NADPH oxidase inhibitor (DPI) reduced NADPH oxidase activity and O_2_^−^ production. The cell proliferation and migration of AFs derived from apoE(-/-) mice were significantly inhibited, further suggesting that p47phox may promote the formation of atherosclerosis through participation in the O_2_^−^ generation. Given these results and previous evidence [26], we postulated that fibroblast proliferation and migration was ROS dependent, inhibition of ROS production in adventitial fibroblasts may be a potential means of attenuating the direct contribution of adventitial fibroblasts to atherosclerosis-related hyperplasia.

We therefore used p47phox specific siRNA to knockdown the expression of p47phox in AFs. As a result of p47phox inhibition (80% of inhibition), NADPH oxidase activity was dramatically decreased with only 1/3 remaining activity compared to control. Both of the proliferation and migration of AFs was attenuated to 50% of control level, suggesting that p47phox plays an important role in regulating NADPH oxidase activity and ROS production. Down-regulation of p47phox level by specific siRNA could be an alternate approach to attenuate the development of atherosclerosis. Our future endeavor will focus on the potential application of p47phox siRNA in both small and large animal models.

## Conclusion

In summary, our data show that p47phox is highly expressed in aorta in hyperlipid fed apoE(-/-) mice. The expression of p47phox increases and spreads from adventitia to intima with continuous hyperlipid diet. The high level of p47phox is associated with enhanced NADPH oxidase activity, ROS generation, and more aggressive proliferation and migration of AFs. Down-regulation of p47phox by siRNA results in attenuated AFs function, suggesting a critical role of p47phox in AFs triggered atherogenesis. The present study not only adds new insights into the mechanism of atherogenesis but also suggests a new candidate target for the treatment of atherosclerosis.
